# Dental Abscess on POCUS: A Retrospective Case Series Analysis

**DOI:** 10.24908/pocusj.v11i01.19441

**Published:** 2026-04-22

**Authors:** Eric Scheier, Barak Gold

**Affiliations:** 1 Pediatric Emergency, Kaplan Medical Center, Rehovot, Israel; 2 Faculty of Medicine, Hebrew University of Jerusalem, Israel; 3 Department of Pediatrics, Kaplan Medical Center, Rehovot, Israel

**Keywords:** Abscess, POCUS, Dental, Periodontal, Periapical, Point of care ultrasound

## Abstract

**Background:**

Point of care ultrasound (POCUS) has proven utility in confirming the diagnosis of skin and soft tissue abscesses. We studied the characteristics of 50 dental abscesses on pediatric POCUS examinations and their outcomes.

**Methods:**

This was a convenience sample of cases collected in the pediatric emergency department from December 2020 to December 2024. All included patients were examined by an oromaxillofacial specialist and hospitalized. All cases had POCUS imaging performed at initial assessment by the first author.

**Results:**

The median age of the patients was 7 years (IQR = 5 years), and 31 (62%) were male. Forty (80%) received antibiotic prior to arrival. Thirty-five (70%) presented within 3 days from onset of pain, and 36 (72%) were referred to the pediatric emergency department by a dentist for oromaxillofacial evaluation. Four children (8%) experienced spontaneous drainage while inpatient, and eleven children underwent surgical drainage (22%). There was a significant correlation between maximal abscess height from bone and the need for surgical drainage (p = 0.011). A cutoff of 4.5 mm yielded a sensitivity of 72.7% and specificity of 69%, indicating that abscesses measuring greater than 4.5 mm are more likely to need surgical drainage.

**Conclusions:**

This was the first known study to describe POCUS findings in children determined to have dental abscess by an oromaxillofacial specialist and hospitalized for treatment. POCUS may have utility in identifying dental abscess, and may be useful in following the progress of abscesses under treatment. The majority of dental abscesses will resolve with antibiotic therapy, but abscesses above 4.5 mm may require drainage early in the course of treatment.

## Introduction

Point of care ultrasound (POCUS) has proven utility in confirming the diagnosis of skin and soft tissue abscess. Limited evidence suggests that skin and soft tissue abscesses with a diameter greater than 1.3 cm are likely to fail medical management [1]. Further, POCUS can differentiate between odontogenic fascial space cellulitis and abscess [2]. The utility of POCUS in guiding therapy of pediatric dental (periapical and periodontal) abscesses is unclear. Here, we describe our experience using POCUS in the pediatric emergency department. We evaluated children with dental abscess in order to determine whether there were characteristics of these POCUS examinations that are associated with subsequent drainage.

## Methods

This study was a retrospective review of POCUS examinations acquired at a tertiary care pediatric emergency department with 27,000 visits annually. Our pediatric emergency department receives children from birth to 18 years of age.

Children with odontogenic facial swelling are referred to the pediatric emergency department by their pediatrician or dentist to confirm a diagnosis of abscess and, if confirmed, inpatient treatment. In our facility, children with odontogenic facial swelling are examined in the pediatric emergency department and followed inpatient by an oromaxillofacial specialist. If the specialist believes the child has failed intravenous antibiotic, drainage is performed under deep sedation. The first author performed all inpatient sedations for these children, as sedation for an oral procedure presents an airway risk that may not be suitable for junior physicians to manage.

Children with odontogenic facial swelling are routinely examined in the pediatric emergency department by the first author with POCUS. We included children of all ages who had an abscess confirmed on POCUS and were then hospitalized. Children who received POCUS but were discharged from the pediatric emergency department were excluded as we had no way to examine outpatient dental records. POCUS was performed by the first author—a pediatric emergency physician with seven years of experience with POCUS—from December 2020 to December of 2024. Measurements were made during review of the images for the study. Hospital charts were reviewed to extract details of emergency department and hospital course. Children who were not seen by an oromaxillofacial specialist and discharged from the pediatric emergency department were excluded. The country-wide Ofek electronic medical record database, which includes all outpatient visits as well as all hospitalizations, was reviewed to verify that the children in our series were not seen in another emergency department for dental abscess.

### Statistics

Descriptive statistics were utilized to summarize the data. Categorical variables are presented as frequencies and percentages, whereas continuous variables are expressed as medians with interquartile range (IQR). Comparisons of categorical variables were performed using the Chi-square test. The normality of continuous variables was assessed using the Shapiro-Wilk test; if the data followed a normal distribution, an independent t-test was used, and if not, the Mann-Whitney U test was employed. Additionally, a receiver operating characteristic (ROC) analysis was conducted. All statistical analyses were performed using SPSS version 29.0, and a p-value below 0.05 was considered statistically significant.

### POCUS Technique

**Figure 1.**
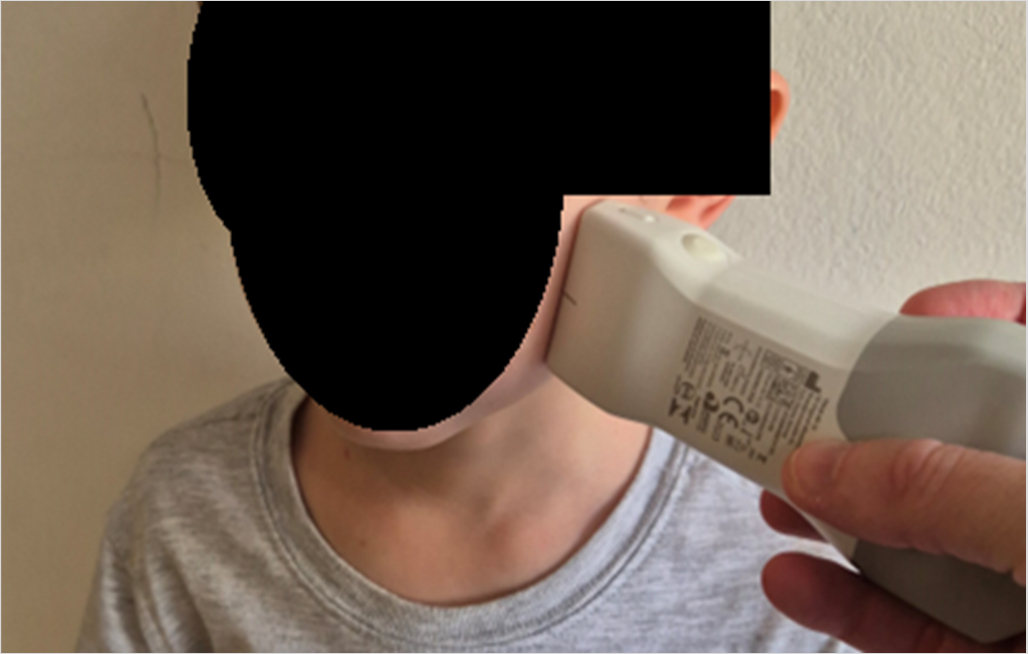
A representation of the sagittal probe placement to identify dental abscess in the maxillary area. The probe should fan through the area of swelling.

**Figure 2.**
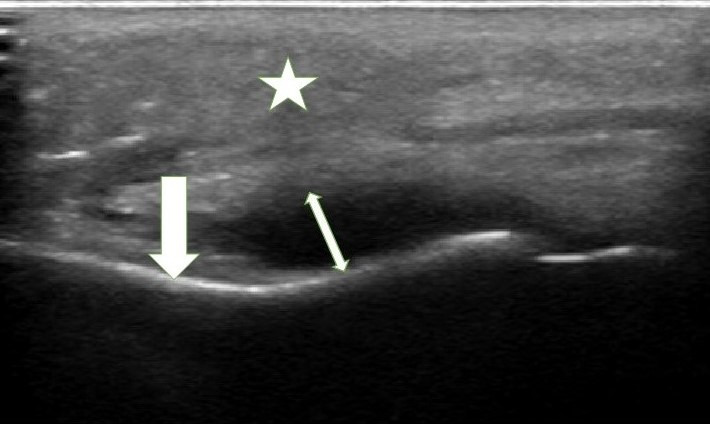
Point of care ultrasound (POCUS) image of dental abscess. Star indicates buccal soft tissue. Single arrow indicates maxillary cortex. Double-headed arrow demarcates the maximum height of an anechoic dental abscess.

All images were acquired with a high frequency linear probe on a Zonare z.1 ultrasound (Mindray, Mahwah, NJ). We started by examining the contralateral side to adjust gain and depth, and to demonstrate to the child that the examination should not be painful. Images were collected in the sagittal plane over the area of swelling (Figure 1). Copious gel was applied and a video sweep in the coronal axis over the area of interest was recorded. Anechoic fluid collections were defined as abscess. After fanning through the abscess, the maximum height of the anechoic fluid pocket from lateral cortex of maxilla or mandible to facial soft tissue was recorded (Figure 2). In contrast with a subcutaneous abscess, which is generally round or ovoid, a periodontal abscess is elliptical within a well-defined anatomic space. In our experience, severity is associated with the height of abscess from bone, which should be identical in coronal and in transverse views. This method of measuring effusion by height rather than volume is commonly used to measure hip effusion on pediatric ultrasound [3]. We therefore recorded abscess height from maxilla or mandible, rather than volume.

## Results

Sixty-one children underwent POCUS examination, of whom eleven were not seen by an oromaxillofacial specialist and were discharged from the pediatric emergency department. None presented after pediatric emergency department discharge to an outside hospital pediatric emergency department. Thus, 50 children with dental abscess treated by an oromaxillofacial specialist were studied. The mean age of this group was 7 years (IQR = 5 years), and 31 (62%) were male. The average duration of pain prior to presentation was just under 4 days, and 40 cases (80%) received antibiotics prior to arrival (Table 1). Seven children had panoramic dental radiography while hospitalized. Four children experienced spontaneous drainage while inpatient and eleven children underwent surgical drainage (22%).

**Table 1.** Characteristics and results of study population. Means are presented as mean with standard deviation.

Variables	N	%
Median age in years (IQR)	7 (5)	
Gender male	31	62
Abscess on the right face	19	38
Abscess over maxilla	27	54
Median pain duration in days prior to POCUS (IQR)	2 (2)	
Antibiotic prior to presentation	40	80
Referred by dentist	36	72
Fever at presentation	11	22
Hospitalized	44	88
Drained in the hospital	11	22

There were no statistically significant associations in white blood cell (WBC), absolute neutrophil (ANC), and C-reactive protein (CRP) counts between operative and non-operative cases (Table 2). All children received intravenous (IV) amoxicillin/clavulanate, except for two who were treated with IV clindamycin due to penicillin allergy. All IV antibiotic courses and drainage were successful. No child re-presented to the pediatric emergency department with recurrent dental abscess. There was no significant association between antibiotic administration prior to pediatric emergency department presentation and either abscess drainage or duration of hospitalization.

**Table 2.** Comparison of age, abscess height and inflammatory markers between children treated medically and children treated with surgical drainage.

Variable	Statistic	No Drainage	Drainage	p-value
Age (years)	Median	6.8	7.0	0.751
	IQR	5.2	5.0	
Maximum abscess height (cm)	Median	0.4	0.5	0.011
	IQR	0.3	0.3	
White blood cell count (cells/microliter)	Median	12,500	9,800	0.449
	IQR	3,309	5,283	
Absolute neutrophil count (cells/microliter)	Median	7,950	7,750	0.879
	IQR	4,499	9,900	
C-reactive protein (mg/dL)	Median	3.4	5.0	0.367
	IQR	7.0	5.8	

There was no statistically significant correlation between duration of symptoms prior to POCUS or between any laboratory parameter and the need for surgical drainage. There was a significant correlation between mean maximal abscess height from bone and the need for surgical drainage (not drained, 0.39 cm; drained, 0.57 cm; *p* = 0.011). Based on the ROC analysis, the area under the curve (AUC) was 0.749, which suggested moderate accuracy in predicting which abscesses might require drainage.

A cutoff of 4.5 mm yielded a sensitivity of 72.7% and specificity of 69%, indicating that there may be an association between abscesses measuring above 4.5 mm and the need for surgical drainage. Including the 11 children who were discharged from the pediatric emergency department and therefore excluded from the initial analysis slightly reduced the model's discriminatory power (AUC decreased from 0.749 to 0.727) and increased the significance level from *p* = 0.012 to *p* = 0.019. Importantly, the optimal cutoff of 0.45 cm remained unchanged, with stable sensitivity and specificity. These findings suggested that the inclusion of milder, non-hospitalized cases did not enhance prediction performance, though it slightly improved generalizability.

Maximal abscess diameters below 0.25 cm had 100% sensitivity for predicting that no surgical intervention was necessary, at the cost of a sharp decline in specificity to 25.6%. While a definitively safe prognostic threshold cannot be established from our data, values below 0.25 cm may support a decision to discharge without intervention if clinical judgment supports that disposition.

## Discussion

Prior small case series in the dental literature have shown that ultrasound can detect odontogenic abscesses [4]. However, this is the first study to describe POCUS findings in children with dental abscess, determined by a pediatric emergency physician. Moreover, all children in our study had a POCUS diagnosis confirmed by an oromaxillofacial specialist, and all were followed through hospitalization. Our study demonstrated that a cutoff of approximately 0.45 cm yields a sensitivity of 72.7% and specificity of 69%, indicating that abscesses measuring 0.45 cm or greater may be associated with need for surgical drainage.

Prior research suggests that a skin abscess diameter above 1.3 cm or depth greater than 0.4 cm is likely to fail antibiotic therapy [1]. While odontogenic periapical abscesses are usually apparent on physical examination, clinical signs alone may not reliably distinguish between cellulitis and true abscess formation in odontogenic infections [2,5]. There is no guidance in the literature regarding the need for drainage of odontogenic abscesses.

Several of our patients underwent panoramic dental radiography. Panoramic radiography can evaluate the dental root and surrounding bone and can demonstrate focal apical lucencies associated with infections originating from the root apices [6,7]. Ultrasound, in contrast with conventional radiographic imaging, has no radiation. Several studies of adult patients with facial pain and swelling have found ultrasound to be more sensitive for abscess than panoramic radiography [8,9]. Barton et al. described two techniques to improve clarity of the image: the first was to have the patient fill their mouth with water and perform a “cheek puff” to push the water between gingiva and buccal mucosa, simulating a water bath. The second was to have the patient use their tongue to point to the area of tenderness [10].

Previous studies have shown that inflammatory markers, such as WBC count and CRP, are frequently elevated in odontogenic abscesses, with elevations less marked in pediatric patients compared to adults [11]. Our study did not find a significant difference in inflammatory markers between children treated surgically (i.e., with drainage) or medically. POCUS allows for early identification of abscess without bloodwork, enabling early treatment. There is a lack of evidence regarding the use of systemic antibiotics in the pediatric population [12,13]. However, early treatment in the outpatient setting may reduce hospitalization [14]. Early treatment is important to prevent dehydration that may result from dental pain and more serious sequelae such as sepsis that has been reported in pediatric dental abscess [15,16]. Thus, early POCUS may obviate the need for laboratory evaluation and systemic antibiotics, and may inform the need for early drainage.

### Limitations

This was a single-center retrospective convenience sample collected by a single physician. While POCUS of a periodontal abscess is a straight-forward examination, we cannot exclude inter-operator variability. POCUS was performed at point of contact and not when the abscess was opened, which in most cases was during hospitalization. The abscess was fanned through in only the sagittal orientation rather than in two orthogonal planes, and height of abscess from cortex rather than abscess volume was measured. The potential space that might contain a dental abscess is limited by the smaller potential space than exists for other subcutaneous abscesses. Because of this, we saw little utility in a second view. While our data can help guide clinical judgment alongside other clinical and laboratory findings, the results are limited by the small sample size, the single-center, retrospective design, and operator-dependent ultrasound measurements. Additional prospective, multicenter studies are needed to validate and refine these recommendations.

## Conclusion

POCUS can be useful for identifying dental abscess and may be useful in following the progress of abscesses under treatment. A POCUS-determined abscess diameter of 4.5 mm is associated with the need for surgical drainage. Larger, prospective studies are required to determine which dental abscesses will require surgical drainage as opposed to medical management, either inpatient or outpatient.
